# Obstetric anal sphincter injuries after episiotomy: systematic review and meta-analysis

**DOI:** 10.1007/s00192-016-2956-1

**Published:** 2016-02-19

**Authors:** Tina Sara Verghese, Rita Champaneria, Dharmesh S Kapoor, Pallavi Manish Latthe

**Affiliations:** 1 Department of Obstetrics and Gynaecology, Birmingham Women’s NHS Foundation Trust, Edgbaston, B15 2TG UK; 2 Birmingham Clinical Trials Unit, University of Birmingham, Edgbaston, UK; 3 Royal Bournemouth and Christchurch Hospital NHS Trust, Bournemouth, UK

**Keywords:** Obstetric anal sphincter injury, Mediolateral episiotomy, Spontaneous vaginal delivery, Perineum

## Abstract

**Introduction:**

There is conflicting evidence on whether mediolateral episiotomy (MLE) reduces the risk of obstetric anal sphincter injuries (OASI) in spontaneous vaginal deliveries (SVD).

**Objectives:**

A systematic review was undertaken to compare rates of OASI amongst women who had undergone mediolateral episiotomy versus those who did not.

**Methods:**

ᅟ

**Search strategy:**

Electronic searches were performed in literature databases: CINAHL, Cochrane, EMBASE, Medline and MIDIRS from database inception to July 2015. Studies were eligible if MLE was compared to spontaneous tears and if OASI was the outcome of interest.

Two reviewers independently selected and extracted data on study characteristics, quality and results. We computed events of OASI in those who did and did not have an episiotomy from individual studies and pooled these results in a meta-analysis where possible.

**Main results:**

Of the 2090 citations, 16 were included in the review. All were non-randomised, population based or retrospective cohort studies. There was great variation in quality amongst these studies. Data from 7 studies was used for meta-analysis. On collating data from these studies where the majority of women (636755/651114) were nulliparous, MLE reduced the risk of OASI (RR 0.67 95 % CI 0.49-0.92) in vaginal delivery.

**Conclusion:**

The pooled analysis of a large number of women undergoing vaginal birth, most of who were nulliparous, indicates that MLE has a beneficial effect in prevention of OASI. An accurately given MLE might have a role in reducing OASI and should not be withheld, especially in nulliparous women. Caution is advised as the data is from non-randomised studies.

## Introduction

In recent years, the rate of third and fourth degree perineal tears have increased to approximately 5.9 % of deliveries in England among nulliparous women [1]. This has the potential to cause long-term physical conditions like anal incontinence and its sequelae. A perineal tear is usually the consequence of inadequate space for the head to deliver or rigidity of the perineum. The severity of this tear may also be related to the degree of control exercised at the time of birth, rapidity of the delivery and interventions used at the time of birth [2].

A median episiotomy is known to increase the risk of obstetric anal sphincter injuries (OASI)[3]. A mediolateral episiotomy (MLE) is a surgical incision given between 45-60 degrees from the midline at the time of crowning to widen the introitus [4,5] The accuracy of the angle at which the episiotomy is performed, the length and depth of the episiotomy and the distance of the incision point of the episiotomy from the midline have all been shown to be influential in determining the incidence of OASI [6,7]. A large retrospective cross sectional study conducted in United Kingdom found that women who delivered without episiotomy were 1.4-1.5 times more likely to sustain an OASI [8]. In contrast, other studies have failed to demonstrate a benefit of the routine use of episiotomy [9,10]. Episiotomy has been shown to be protective in instrumental deliveries in large studies [1,11]. National Institute of Health and Care Excellence (NICE) has recommended the use of episiotomy in instrumental deliveries [12] whereas the American College of Obstetricians and Gynecologists (ACOG) has not. [13]. This is due to an increased incidence of perineal pain and dyspareunia. [14].

The Cochrane systematic review suggests that there is no role for routine episiotomy in spontaneous vaginal delivery[9]. However, the Cochrane systematic review included both median and mediolateral episiotomy studies as well as women of all parities. We undertook a systematic overview of the current available literature from key medical databases to study specifically whether women who had mediolateral episiotomy had less risk of OASI as compared to women who sustained perineal tears during spontaneous vaginal delivery.

## Methods

This meta-analysis was performed in accordance with widely recommended methods (PRISMA)[15]. We considered this study to be exempt from Ethics Committee approval.

## Identification of studies

The following bibliographic databases were searched for relevant citations, from database inception to July 2015: CINAHL, Cochrane, EMBASE, Medline and MIDIRS. Our search strategies consisted of MeSH subheadings, text words and word variations for the concepts of ‘birth’, ‘episiotomy’, ‘perineal tear and injuries’ and ‘obstetric anal sphincter injury’. The basic search strategy was adapted to suit the database being searched. The search was restricted to ‘humans’ and ‘females’. Bibliographies of relevant primary articles were also searched in order to identify any articles missed by the electronic searches. No language restrictions were applied.

## Study selection and data extraction procedures

Studies were selected following a two-step process. Firstly, the citations identified by the electronic bibliographic database searches were screened, based on their titles and abstracts. Full text papers of eligible abstracts were retrieved. Once full text papers had been located, we determined whether they fulfilled our predetermined inclusion criteria:

- **Population**: Women undergoing non instrumental vaginal birth
- **Intervention**: Mediolateral episiotomy (MLE)
- **Comparator**: No episiotomy or spontaneous perineal tear
- **Outcome**: Third or fourth degree perineal tears/ Obstetric anal sphincter injury (OASI).
- **Study designs**: All except case series or reports

Studies with the following were excluded: midline episiotomy, instrumental or operative vaginal deliveries, no data on OASI. If a study reported rates of OASI for both instrumental and non-instrumental deliveries but presented them separately, the paper was included and data for the non-instrumental delivery arm was used.

Two reviewers independently assessed the full text papers to determine if they met the above criteria. Any disagreements surrounding the eligibility of a paper, was either solved through consensus or arbitration by a third reviewer (PML). Data from included manuscripts were extracted onto a pre-designed pro-forma. Data was collated on study characteristics, including methods of recruitment, patient characteristics, details of both spontaneous tears and episiotomy, outcomes and results. We contacted primary authors via email for any further information that was required. Studies fulfilling the inclusion criteria, were included in the systematic review, and those where the data could be abstracted into a 2 × 2 table were included in the meta-analysis.

## Methodological quality assessment and data synthesis

The methodological quality of all the papers fulfilling the inclusion criteria was assessed. The Newcastle-Ottawa Quality Assessment Scale was used for observational studies. The quality checklist utilised awards one star as maximum for all items except comparability where it can award a maximum of two stars. The quality of the article is scored based on the selection, comparability and outcomes. An arbitrary score based on the assumption of equal weight of all items included in the Newcastle-Ottawa Scale. This was used to give a quantitative appraisal of overall quality of the individual studies. The score ranged from 0 to 9, with a score of either 0 or 1 for each item.

Data on the number of women with/without episiotomy and with/without OASI, were used to populate 2 × 2 tables and generate relative risk ratios. Relative risks from individual studies were meta-analysed using a random effects model for analysis [16]. Subgroup analyses were performed by dividing studies according to parity. Studies were categorised as involving ‘nulliparous’ women, who were included in the top plot (Fig. 2), or multiparous women, or studies that did not state the parity of women (Fig. 2), which were included in the lower plot, entitled ‘combined’. Heterogeneity was evaluated graphically using forest plots and statistically using the I^2^ statistic to quantify heterogeneity across studies [17]. Statistical analysis was performed using Review Manager 5.1 (Copenhagen: The Nordic Cochrane Centre, The Cochrane Collaboration 2011).

## Results

**Fig. 1:**
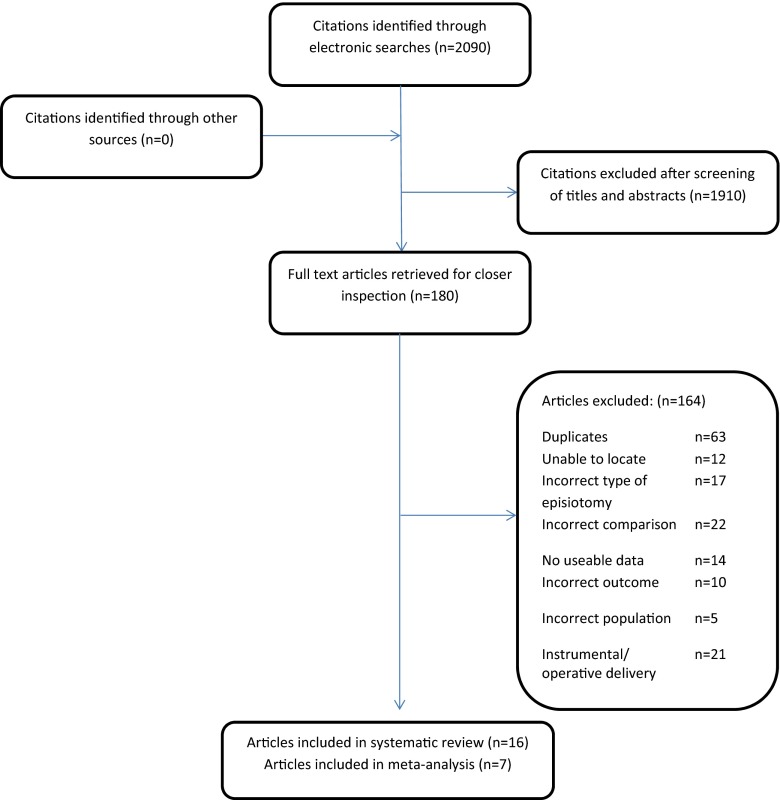
The flow of literature search

**Fig. 2:**
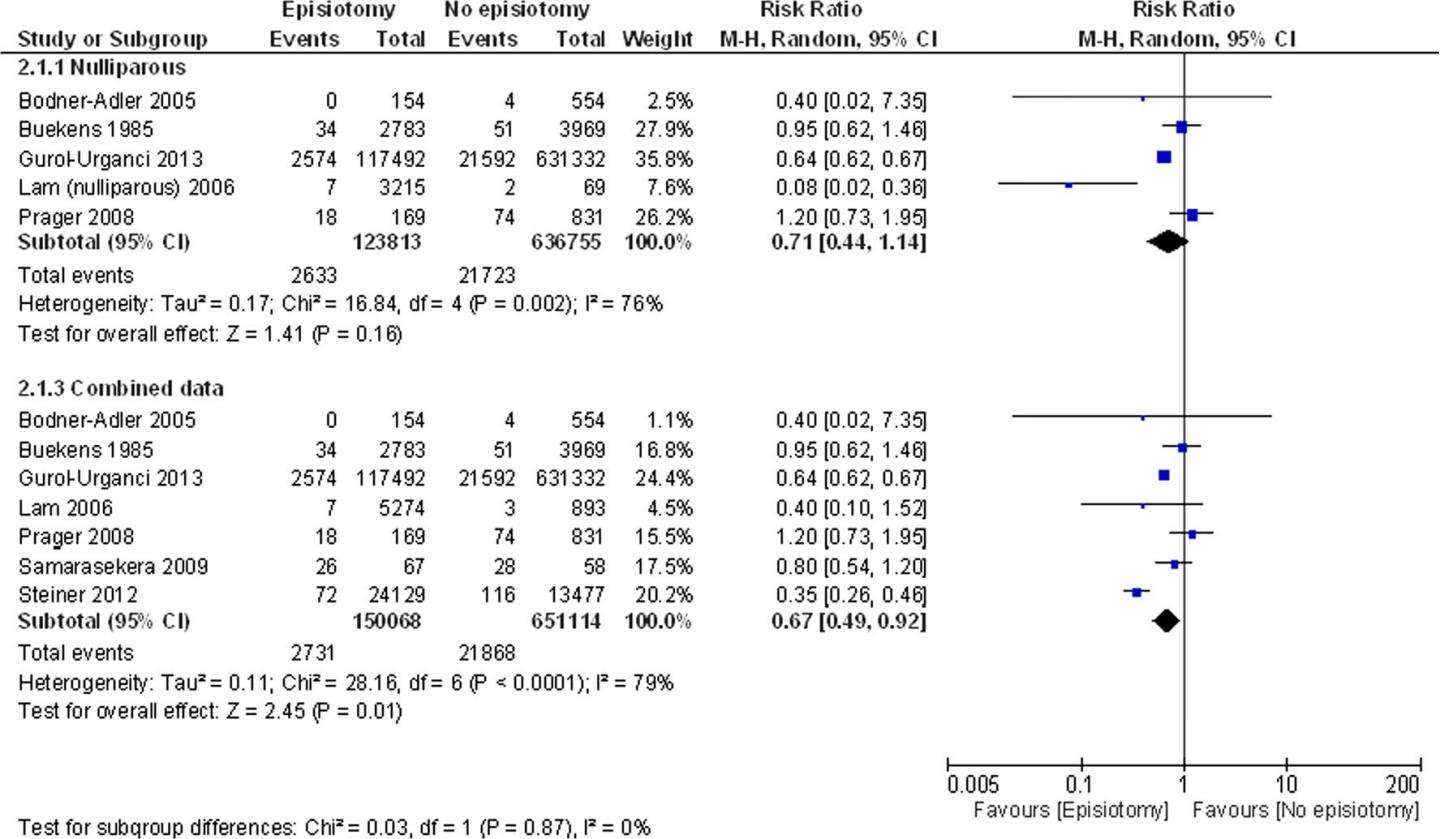
The risk benefit relationship between the administration of MLE and occurrence of OASI based on parity

Figure 1 shows the flow of literature from identification of citations through to inclusion of the studies in the review. Sixteen non-randomised studies were included in our review and data from 7 of these were presented in a format that could be used in the meta-analysis. (Fig. 2).

Table 1 summarises the characteristics of the studies included in this review. The population of interest in 6/16 studies were nulliparous women undergoing a spontaneous vaginal delivery. In the studies that looked at mixed parity (i.e. nulliparous and multiparous) wherever possible we endeavoured to separate data based on parity. There was a wide variation among the sample size of patients included in the studies (range 125-1673442). The outcomes reported by included studies were severe perineal tears (third or fourth degree) and some also reported on postnatal anal incontinence.

**Table 1:** Characteristics of studies included in the OASI after episiotomy

Author, year, country	Study design	Age	Sample size	Confounders in the study that were adjusted	Intervention	Control
*Characteristics of articles included in the systematic review and meta*- *analysis*						
Gurol- Urganci [1] 2013, UK	Retrospective Cohort study	15-45 years	1035253	Ethnicity, Socio-economic deprivation measured using index of multiple deprivation, Prolonged labour, Birth-weight	Mediolateral episiotomy (MLE)	Spontaneous vaginal tear
Prager [30] 2008, Sweden	Retrospective cohort study	25- >/= 42 years	2000	Birthweight, duration of second stage, use of oxytocin, perineal protection technique	MLE	Women who had no episiotomy
Bodner-Adler[31] 2004, Austria	Retrospective cohort study	Median age at delivery 26 years	725	Use of oxytocin, epidural analgesia, maternal position at delivery, length of first and second stage of labour, birth weight	MLE	No control
Buekens [32] 1985, Belgium	Observational cohort study	Not mentioned	21278	Birthweight , parity	Mediolateral episiotomy	Women without episiotomy
Lam [33]2006, Hong Kong	Retrospective cohort study	Mean – 29 years	6222 (3312 primiparous and 2910 multiparous)	No other risk factors were taken into account apart from parity	Mediolateral episiotomy	Spontaneous vaginal tears
Samarasekera [24] 2009, UK	Retrospective cohort study All women who suffered third degree tears between 1981-1993 found through delivery records	Mean 26.9 years	125 (54 – had third degree tears, 71 – had an uncomplicated vaginal delivery)	Birth weight, length of second stage, maternal age, parity	.(MLE data extracted)	Women who had an uncomplicated vaginal delivery during 1981 – 1993
Steiner [34] 2012, Israel	Retrospective cohort study	Not mentioned	168,077	Confounders controlled for macrosomia, non-reassuring fetal heart rate, occipito -posterior position and shoulder dystocia	MLE	Without episiotomy
*Characteristics of articles included in the systematic review*						
Andrews [35] 2006, UK	Retrospective cohort study	Not mentioned	241	Confounding factors not adjusted	MLE	No control
Jango, [11] 2013, Denmark	Retrospective Cohort study	Median – 28 years	214256	Birth weight, epidural, Induction of labour, Oxytocin augmentation	MLE	No control
Baghestan[36] 2010, Norway	Retrospective cohort study	Mean – 24 years	1,673,442	Maternal age, birth-order, Previous caesarean birth, birth weight, ethnicity, induction of labour, analgesia	Mediolateral episiotomy	No control
Ampt[37] 2013, Australia	Retrospective cohort study	20- 40 years	528,846	Confounding factors were not adjusted	Mediolateral episiotomy	No control
Revicky [8] 2010, UK	Retrospective cross sectional study	20- 41 years	10314	Type of augmentation, epidural, Birth weight	MLE	No episiotomy
Shihadeh [38] 2001, Jordan	Retrospective cohort study	Not mentioned	17559 (Primiparous = 3875, Multip = 13684)	To avoid confounding factors, the study analysed a sub sample which included vertex vaginal deliveries and stratified further based on birthweight	MLE	Without episiotomy
Angioli[39] 2000, USA	Retrospective cohort study	Mean 25.5 +/- 6.4 years	Primiparous 19360 Multip - 30850	Ethnicity, birth weight, maternal age , parity	MLE data extracted	No control
Mora-Hervas [40] 2015, Spain	Cohort study	Not mentioned	938	Not mentioned	MLE	Spontaneous vaginal tears
Twidale, [23] 2013, Australia	Retrospective cohort study	Not mentioned	7314	Birth weight, epidural, induction of labour	MLE	No control

Table 2 summarises the quality of the included studies. There was variation in each of the quality domain questions: consecutive recruitment, description of how angle of episiotomy was measured, adequate follow-up, and validated outcome assessment (e.g. adequate clinical examination to diagnose OASI). On the Newcastle-Ottawa Quality Assessment Scale most had scores between 4-8, suggestive of a moderate risk of bias.

**Table 2:** Quality assessment of cohort studies utilising the Newcastle Ottawa quality assessment scale

STUDY	SELECTION				Comparability		Outcome			SCORE (out of 13)
	Representativeness	Selection of non-exposed cohort	Ascertainment of exposure	Outcomes of interest	Controls	Additional factors	Assessment of outcome	Follow-up	Adequacy of follow-up	
Gurol-Urganci[1] 2013	*	x	*	*	*	x	* record linkage	x	x	5
Prager[30] 2008	*	x	*	*	*	*	* record linkage	x	x	6
Bodner- Adler [31]	*	x	*	*	*	*	*	x	x	5
Buekens[32] 1985	*	x	*	*	*	*	*	x	x	6
Lam [33] 2006	*	*	*	*	*	x	* record linkage	x	x	6
Samarasekera,[24] 2009	*	*	*	*	*	*	*	*	x	8
Steiner[34] 2012	*	*	*	*	*	*	*	x	x	7
Andrews[35], 2006	*	x	*	*	x	x	*	x	x	4
Jango [11] 2013	*	*	*	*	*	*	*	x	x	7
Baghestan[36] 2010	*	*	*	*	*	*	*	x	x	7
Ampt,[37] 2013	*	x	*	*	*	*	*	x	x	6
Revicky, [8] 2010	*	x	*	*	*	*	*	x	x	6
Shihadeh,[38] 2001	*	*	*	*	*	*	*	x	x	7
Angioli, [39] 2000	*	*	*	*	*	*	*	x	x	7
Mora-Hervas [40] 2015	*	*	*	*	*	x	*	*	x	7
Twidale, [23] 2013	*	x	*	*	*	*	*	x	x	6

*Indicates that a feature is present; x, that a feature is absent. But for comparability by design this checklist awards a maximum of two stars (**), one (*) or none if the feature is completely absent (x)

Figure 1 depicts the risk benefit relationship between the administration of MLE and occurrence of OASI based on parity. In the meta-analysis of women who had spontaneous vaginal delivery, the relative risk of OASI is reduced with MLE (RR 0.67 95 % CI 0.49-0.92, p = 0.01, *I*^*2*^ = 79 %). The forest plot, however, showed no significant difference in OASI in nulliparous subgroup (0.71 RR 95 % CI 0.44–1.41, p = 0.16, *I*^2^ = 76 %). The I^2^ statistic was high (70-80 %) indicating true heterogeneity between studies.

## Discussion

The protective effect of episiotomy against OASI was noted in the meta-analysis (RR 0.67 95 % CI 0.49-0.92) of 7 studies. The number needed to treat was 65 i.e. 65 additional episiotomies would have to be given to prevent one additional OASI. The pooled results from 5 studies with nulliparous women only, suggested that the rate of OASI is not different in those who had MLE versus those who sustained perineal tear although there was trend towards protection (RR 0.71 95 % CI 0.44-1.14). Failure to reach statistical significance could be due to the lesser number of women in this subgroup.

We conducted an extensive search strategy without language restrictions. The systematic review was conducted by two independent reviewers and disagreements resolved by a third reviewer. We followed a priori protocol and used valid data synthesis methods. The Newcastle –Ottawa Quality scale was utilized to assess the quality of included studies. Studies that were comparable in terms of patient characteristics, methods and outcomes of interest were collated but we could not include 9/16 studies, as we could not get the detailed numbers to include in the meta-analyses. We used the random effects model to compensate for the different biases introduced by the non-randomised studies included and to reduce the risk of over exaggeration of the effect of intervention [18]. Most of the studies have mentioned confounding factors and have endeavoured to adjust for these factors such as ethnicity, maternal age, length of second stage of labour, epidural analgesia and birth weight. Other potential confounders include manual perineal support, perineal massage, warm compress and other perineal management techniques that aim to reduce the rates of perineal trauma. We have included details of perineal techniques if any were discussed in the individual studies in Table 1. There were few studies that reported OASI in both nulliparous and multiparous women together and in these studies we were unable to categorise the data by parity. Authors of studies were contacted and data were shared where possible. Some studies did not provide sufficient detail on whether the control group had spontaneous mild perineal tears or had an intact perineum. The majority of studies gave either inadequate or no details on the practical execution of MLE. Only one study measured the angle of the MLE to ascertain whether it was accurately performed. Therefore we had to rely on the stated intent of the authors as to the type of episiotomy.

## Our findings in context of the existing literature

Our systematic review aimed to compare effect of MLE versus spontaneous tears in OASI. In contrast, the Cochrane systematic review by Carrolli et al compared restrictive versus routine use of episiotomy. Restrictive episiotomy showed no difference in severe vaginal/perineal trauma (RR0.92, 95 % CI 0.72-1.18). The RCTs included in this systematic review included median as well as mediolateral episiotomy [19]. McLeod et al found a higher incidence of short-term perineal pain in the restrictive episiotomy compared to the routine episiotomy group [20]. Episiotomy was also found to have a protective effect on quality of life and pelvic floor symptoms at one-year follow up [21].

Revicky et al in 2010, reported on a cohort of 10,000 vaginal deliveries where women giving birth without a MLE were 1.4 times more likely to experience OASI (95 % CI 1.021-1.983) [8]. In 2015 they reported that on multivariate regression analysis of 40,777 births, OASI was found to be strongly associated with risk factors such as higher birth weight, instrumental delivery, primiparity and maternal age. MLE reduced the risk of OASI by 4.55 times (OR 4.55, 95%CI 3.7-5.6, p < 0.0001) [22].

Laine et al, reported a reduction in OASI from 4.03 % (285 of 7,069) to 1.17 % (42 of 3,577) (p < 0.001). This was attributed to the use of hands-on technique during the second stage of labour. The number of episiotomies had also however, increased from 13.9 % to 21.1 % [2], and it would be difficult to ascribe weightage to each of this interventions [2]. Twidale et al reported a significant correlation between increasing MLE use from 12.56 to 20.10 % and a reduction in OASI rates over a 5 year period in a retrospective observational study [23]. In a multiple logistic regression analysis, MLE was shown to be a significant protective factor against development of OASI compared to spontaneous tears [24]. Gurol-Urganci et al reported that the rate of OASI tripled from 1.8 % to 5.9 % from 2000 to 2012 in the United Kingdom, based on the analysis of the hospital episode statistics (HES) data [1]. They found that nulliparous women who received MLE were less likely to have OASI (2574/117492) compared to women who did not have one (21592/631332).

## Implications for clinical practice

An episiotomy is defined by variables such as the location of the beginning of the cut, the incision angle along with its length and depth. In a prospective study, an incision angle of mediolateral episiotomy of 60° resulted in a low incidence of anal sphincter tearing, anal incontinence and perineal pain [25]. There is a 50 % relative reduction in risk of sustaining a third degree tear for every 6 degree away from the perineal midline that an episiotomy was cut [26]. Where episiotomy is indicated, the mediolateral technique should be used on the distended perineum, with careful attention to ensure that the angle is 60 degrees away from the midline [27] .

## Implications for further research

This meta-analysis of observational data is the best available evidence on the effect of MLE versus spontaneous perineal tear during non-instrumental vaginal delivery and the resultant OASI rate. There might be ethical objections to a multicentre adequately powered robustly conducted randomised controlled trial to evaluate the effectiveness of use of accurately given mediolateral episiotomy versus spontaneous perineal tear in reducing OASI during the first vaginal birth. The Episcissors-60 delivers a consistent post-delivery angle of 43 degrees [28]. They could be used when performing mediolateral episiotomies and form part of an evaulation of preventative strategy to reduce OASI to be tested in practice [29].
